# Serial Tap Test of patients with idiopathic normal pressure hydrocephalus: impact on cognitive function and its meaning

**DOI:** 10.1186/s12987-021-00254-3

**Published:** 2021-05-06

**Authors:** Samanta Fabrício Blattes da Rocha, Pedro André Kowacs, Ricardo Krause Martinez de Souza, Matheus Kahakura Franco Pedro, Ricardo Ramina, Hélio A. Ghizoni Teive

**Affiliations:** 1 Neurological Institute of Curitiba (INC), Curitiba, Street Jeremias Maciel Perretto, 300, Curitiba, Paraná 81210-310 Brazil; 2 Headache Division and Pain Residence, Neurology Division, Hospital Clinics, Federal University of Paraná, Curitiba, Brazil; 3 Neurology Service, Internal Medicine Department, Hospital Clinics, Federal University of Paraná, Curitiba, Paraná Brazil

**Keywords:** Idiopathic normal pressure hydrocephalus, Tap test, Serial lumbar puncture, Cognition

## Abstract

**Background:**

Idiopathic normal pressure hydrocephalus (INPH) is characterized by gait disturbance, urinary incontinence and cognitive decline. Symptoms are potentially reversible and treatment is based on cerebrospinal fluid shunting. The tap test (TT) is used to identify patients that will benefit from surgery. This procedure consists of the withdrawal of 20 to 50 mL of cerebrospinal fluid (CSF) through a lumbar puncture (LP) after which the symptoms of the triad are tested. Improvement in the quality and speed of gait are already recognized but cognitive improvement depends on several factors such as tests used, the time elapsed after LP for re-testing, and the number of punctures. Serial punctures may trigger similar conditions as external lumbar drainage (ELD) to the organism.

**Objective:**

This study aimed to identify how serial punctures affect cognition to increase the sensitivity of the test and consequently the accuracy of surgical indication.

**Methods:**

Sixty-one patients with INPH underwent baseline memory and executive tests repeatedly following the 2-Step Tap Test protocol (2-STT – two procedures of 30 mL lumbar CSF drainage separated by a 24-h interval). The baseline scores of INPH patients were compared with those of 55 healthy controls, and with intragroup post-puncture scores of the 2-STT.

**Results:**

The group with INPH had lower performance than the control group in all cognitive tests (RAVLT, Stroop, CFT, FAR-COWA, FAB, MMSE, orientation, mental control), except for the forward digit span test (p = 0.707). After conducting LP procedures, the Stroop test (words, colors and errors), RAVLT (stage A1, A6 and B1), and CFT (immediate and delayed R) scores were equal to those of the control group (p > 0.05). The INPH group presented significant improvement after the first puncture in MMSE (p = 0.031) and in the Stroop Test (points) (p < 0.001). After the second puncture, subjects improved in orientation, MMSE, RAVLT (B1), Stroop (points, words, errors) and CFT (IR).

**Conclusion:**

Progressive cognitive improvement occurred over the 2-STT and changes were more significant after the second LP in all cognitive domains except for RAVLT (A7). Encephalic alert system ‘arousal’ seems to participate in early improvements observed during 2-STT. The second LP increased the sensitivity of the drainage test to detect changes in cognitive variables, and consequently improved the quality of the method.

## Introduction

Idiopathic normal pressure hydrocephalus (INPH) is characterized by the classic triad [1] of progressive symptoms: gait apraxia, dementia, and urinary incontinence resulting from reasons that are not fully explained [2]. Cognitive alterations involve executive, attention, memory, and processing speed dysfunctions [3].

Symptoms of INPH can be alleviated with ventriculoperitoneal shunts [4]. To emulate this procedure, tests that promote the temporary removal of cerebrospinal fluid (CSF) are used to evaluate effects on gait and cognition. The most used methods are: a) the lumbar drainage test, known as Tap Test (TT); and b) the continuous lumbar drainage test (CLD). TT has high specificity (73–100%) but low sensitivity (26–79%) [5]. Marmarou et al*.* [5] and Ishikawa et al*.* [6] confirmed CLD to be more sensitive (50–100%) than TT, but it is less popular due to invasiveness and morbidity [7, 8].

Aspects such as the amount of CSF drained and measurements of changes in gait and cognition may influence TT outcomes. There is evidence that the effects of CSF drainage in TT can extend beyond the time needed for CSF to regain its original volume and can last for 24 h or longer [9]. Despite the well-established methods to quantify gait improvement, there are still controversies about cognition improvement with TT [3].

Although memory mechanisms as learning, retention and retrieval are well known [10, 11], research on memory has not fully clarified the mechanisms of forgetting. The theory of interference [12–14] postulates that forgetfulness derives from the interference of one memory over another. There is evidence that the most important mechanisms are proactive interference (PI), in which prior learning affects later learning, and retroactive interference (RI), in which new information may interfere with prior learning. Thus, given the multiple mnemonic systems that are interacting mutually, studies using complex tests such as the Rey Auditory Verbal Learning Test (RAVLT) may help us to understand how this interaction occurs in a clinical condition that affects memory as INPH.

Ishikawa et al. [6] suggest that cognitive and urinary improvements may still occur up to one week after lumbar puncture. Serial lumbar punctures can produce, by analogy, similar physiological effects to CLD, with corresponding effects on gait and cognition. Thus, with multiple punctures, it may be possible that potential later cognitive amelioration can emerge earlier. The aim of present study was to evaluate the cognitive impact of serial tap test (STT) in patients with INPH.

## Material and methods

The protocol was approved by the local regulatory committee for having followed the principles of the Helsinki Convention and its later amendments, as well as Brazilian guidelines disposed by resolution No. 466/2012, all individuals were adequately consented.

## Population

### INPH population

**Fig. 1:**
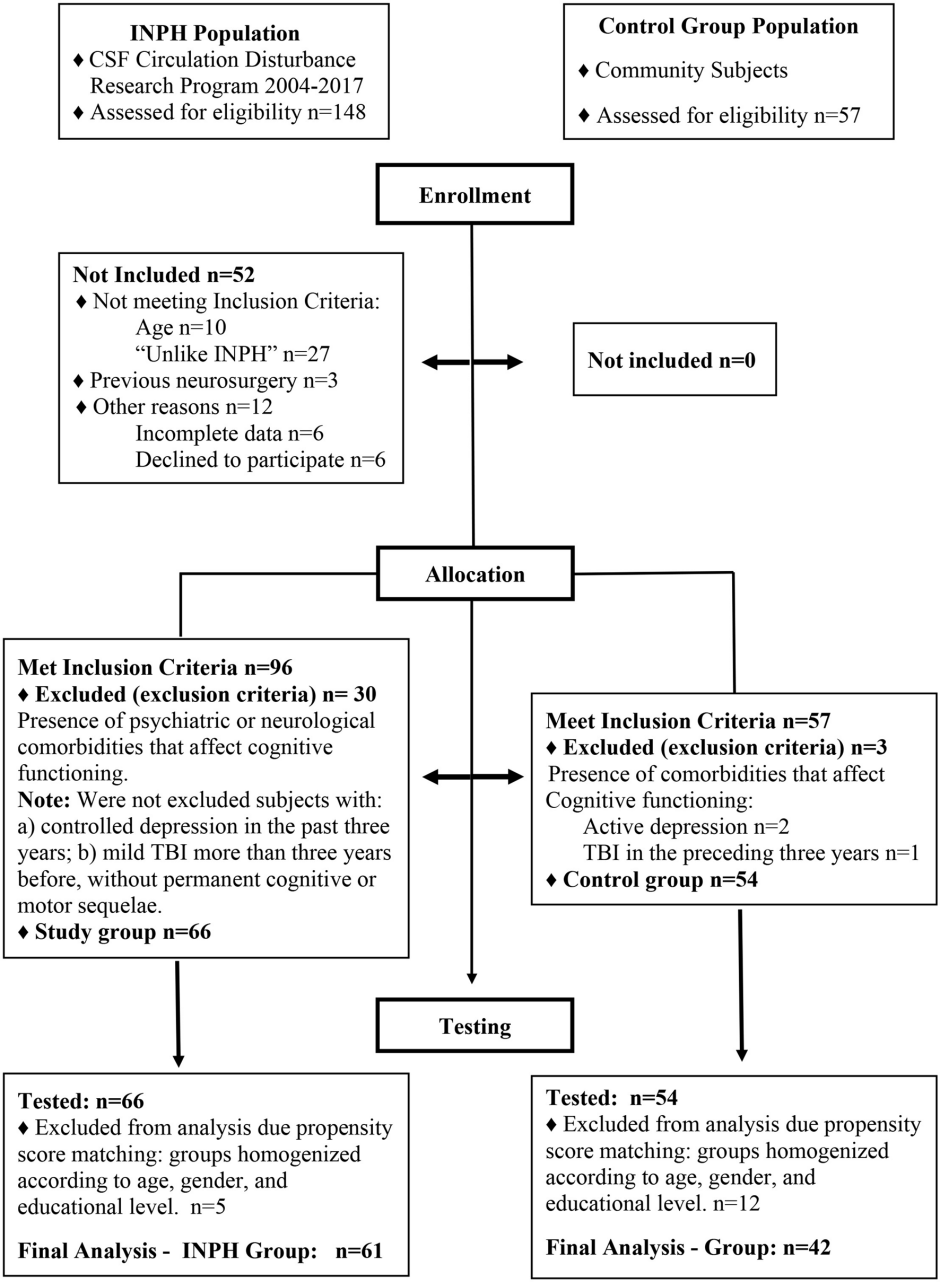
Research flow

Study subjects were initially searched at the CSF Circulation Disturbance Research Program 2004–2017 files. This database contains information on well-established standardized procedures carried out at the Neurological Institute of Curitiba in suspected INPH subjects. Of the hundred and forty-eight subjects suspected of having INPH initially found, ten were not included for being aged 58 years or less, 27 for being classified as “unlikely”, three for having been previously submitted to a neurosurgical procedure and further twelve for refusing to consent with the study procedures and other reasons (e.g.: missing data). Of the remaining 96 individuals with "probable" or "possible" INPH (according to the 2005 Euro-American Consensus) [5] were selected. Of those 96 subjects, 30 individuals were excluded due to comorbidities that could potentially impact cognitive functioning, such as active alcoholism, Parkinson's disease or parkinsonism, Alzheimer's disease, epilepsy, central nervous system [CNS] tumor, other neurological diseases such as vascular dementia, Lewy body disease, and multiple sclerosis; psychiatric disorders such as major depressive disorder, bipolar affective disorder, and attention deficit and cerebrovascular disorders (Fig. 1 and Table 1).

**Table 1:** Evolution over time of the classic HPN features in the INPH group

Variable	n	%	M	SD	Range
Age at onset of symptoms (years)	61		73.2	6.7	
Time between start and STT (months)	61		27.7	26.2	
Age at STT	61		75.5	6.5	58–86
Educational level—years	61		11.5	4.9	2–23
Male	31		50.8		
< One year with symptoms	15	24.6			
> One year with symptoms	46	75.4			
< Two years with symptoms	35	57.4			
> Two years with symptoms	26	42.6			
Gait	61	100			
Cognition	57	90			
Sphincter	45	69.8			
One symptom (gait)	4	6.7			
Two symptoms (*)	21	35			
Complete triad	35	58.3			

Values described in mean (M), standard deviation (SD) and percentages (%). *STT* Serial Tap Test, *n* number of individuals; ≤ : values equal to or less than the cut-off point; > : values higher than the cut-off point; * gait apraxia associated with cognitive or sphincter symptoms

Individuals with a history of depression under control for more than three years or a history of mild TBI without permanent sequelae (cognitive or motor) were recruited (Table 2).

**Table 2:** Associated clinical conditions of the group with INPH

Variable	Sample (N)	n	%
Neurological events^a^		16	26.22
TBI		8	
Stroke		6	
SAH		1	
TIA		1	
Hypertension	57	36	63.2
History of Depression	60	15	25
Diabetes	61	15	24.6
Dyslipidemia	61	11	18
Hypothyroidism	60	7	11.7
Smoking	60	3	5

Data described in original sample number (N), number (n) and percentages (%); *TBI* traumatic brain injury, *SAH* subarachnoid hemorrhage, *TIA* transient ischemic attack. All individuals in the group with stroke [n = 6] presented a time interval between the stroke and STT greater than three years. Three subjects presented lower-impact ischemic stroke without sequelae. One individual had three episodes: in the first he became unable to wear his slippers, in the second there was loss of peripheral vision, and finally right hemiparesis in the third

### Control population

A control group was recruited from the community (patient’s and or hospital staff’s families and from an aged Baptist Church group). They were submitted to an interview about medical, scholarship and functional history, besides current health. Individuals suspected of neurological or psychiatric diseases were not included. An initial group of 57 individuals was selected. Of these, three subjects were excluded because of depression and brain trauma in the preceding 3 years.

### Study final population

**Fig. 2:**
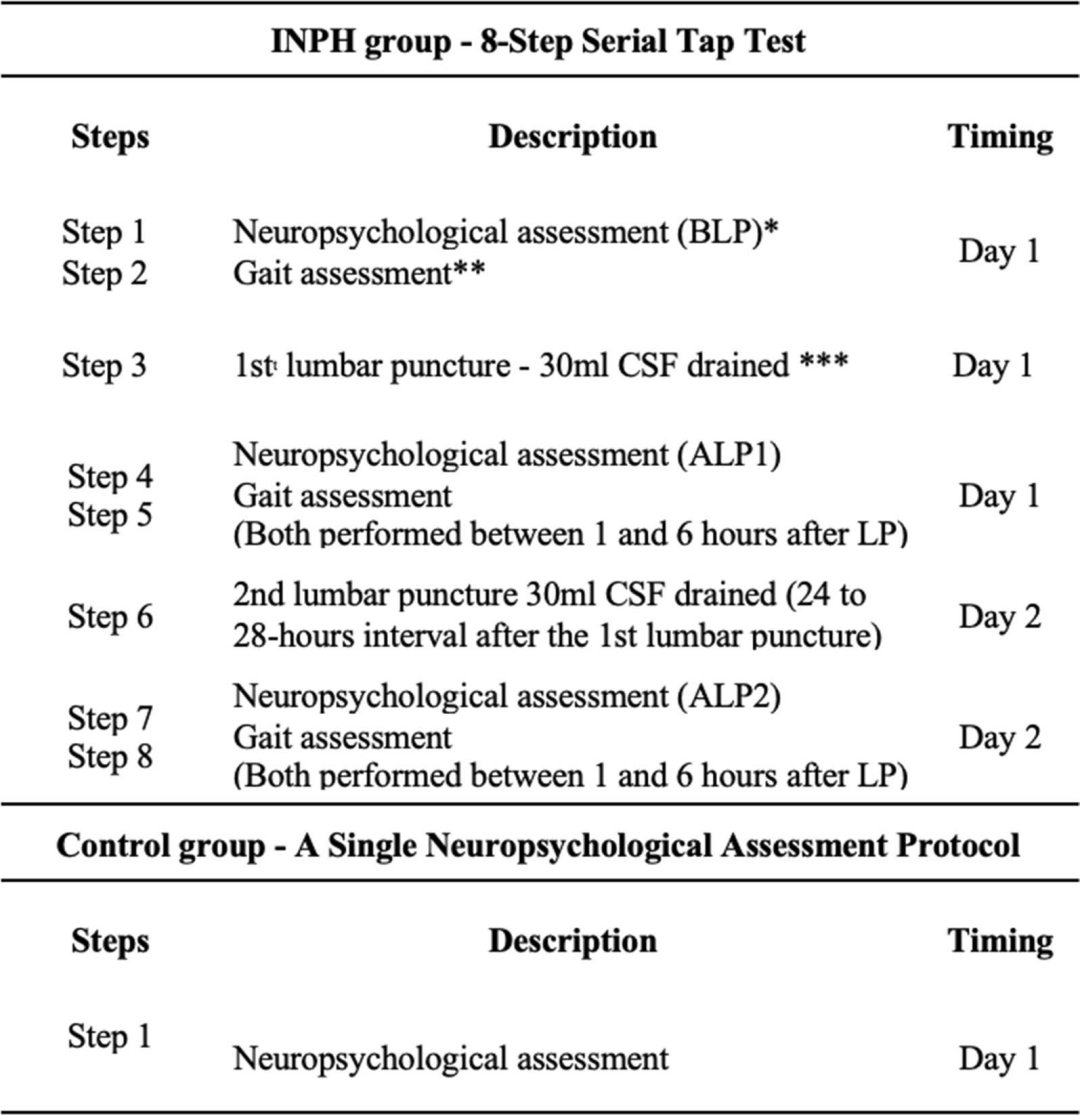
Serial Tap Test Procedure. LP (Lumbar puncture), INPH group (Idiopathic normal pressure hydrocephalus). BLP: step before lumbar punctures; ALP1: step after the first lumbar puncture; post ALP2: step after the second lumbar puncture. * Scores from 1^st^, 4^st^ and 7^st^ INPH group steps were compared with 1^st^ Control group step (Table 3). * Scores from 1^st^, 4^st^ and 7^st^ INPH group steps were compared for detecting changes along the procedure in the INPH group (Table 4). ** See [16] for further details about gait assessment. *** Quincke spinal needle 22G with metallic stylet introduced at an angle of 60º with the skin at the midline. A spinal manometry was conducted to assess the opening pressure

The remaining 66 INPH individuals and 54 control subjects underwent propensity score matching and both groups were homogenized according to age, gender, and educational level. This led to the exclusion of five individuals from the INPH group and 12 individuals from the control group. Data of 61 individuals from the INPH group and 42 from the control group were then compared (Fig. 2).

Procedures

### Tap Test

Patients were admitted to the hospital for two to three days. Two lumbar punctures were performed at intervals of at least 24 h apart (minimum 24 h and maximum 28 h, to guarantee total LCR turnover) and 30 mL of CSF (M: 26 ± 1.2), was withdrawn in each, as described by Adams [1]. CSF opening pressure was measured and a CSF sample was sent for laboratory analysis. The first LP was preceded by cognitive and gait examinations performed by a team of three neuropsychologists and four neurologists, respectively. After being examined for excluding post-puncture pain or headache [9] and between two and six hours after each LP, INPH subjects were tested again for both gait and cognitive examinations. Gait and cognition were never assessed within an interval shorter than one hour after the LP (M = 4,4 ± 2,5). Neither persistent headache nor persistent pain after lumbar puncture were reported by individuals in this series, and further information regarding this issue can be found in a recent paper of our group [15].

To minimize interindividual bias, the neuropsychology team was constantly trained on study procedures. Furthermore, study data were discussed weekly and investigators remained blind to investigation subjects' diagnosis during study procedures. Gait protocol has been described elsewhere [16] and will not be discussed in this paper.

### Neuropsychological examination

The neuropsychological assessment was performed during hospitalization. The average duration of the cognitive protocol was one hour and thirty minutes. Some data was not collected from patients with visual or motor impairment. The cognitive tests used were: Orientation (self-awareness, orientation in place, time and current context). Mental Control (usual and unusual activities: to speak the days of the week, the months of the year, to count (e.g.: 1–20) and after this, all the same activities are repeated in backward), FAR-COWA (The examinee must produce orally as many words as possible beginning with a specified letter during one minute (F-A-R) and thereafter he is asked to produce as many animal names as possible within a one-minute interval); Rey Auditory Verbal Learning Test (RAVLT): This is an episodic memory test: a 15-word list is read aloud (A list) for five consecutive trials (A1–A5). After trial A5, a distractor 15-word list is presented once and the examinee needs to learn it (B1); immediately after this, the examinee is asked to recall the A list (A6—immediate recall) and twenty minutes later again (A7—delayed recall); Rey Complex Figure Test (CFT): A visuospatial memory test: the examinee needs to learn a new complex figure by copying it. After a three-minute interval, he is asked to reproduce the figure as similar as possible without looking at it and thirty minutes later again); Digit Span Test (WMS-R): A two-step working memory test: 1—the examinee is asked to repeat series of numbers in the same order that they are presented by the examiner; the quantity of digits increases progressively (digit span forward); 2—the examinee is asked to repeat similar series but in backward (digit span backward). In our protocol both parts are scored separately; Stroop test (University of Victoria Version): This is an attention test: the examinee is presented to three cards. In the first card, there are 24 colored dots (blue, red, yellow, green). The subject is required to name the colors in which the stimuli are printed as fast as possible. In the second part, neutral words (when, where, what) are printed in the same colors of the dots and the subject needs to name the colors while ignoring the words. In the third part, the words blue, red, yellow and green are printed in conflicting colors (e.g.: the word “blue” printed in red color). Individuals are asked to name the colors printed again while avoid reading the words (name of colors). Scores are calculated based on the speed to perform the tasks and on the number of errors only in the third part; Mini Mental Status Examination (MMSE): This is a 30-point scoring test composed of “Introduction” section—items of orientation, memory and attention (21 points); “Materials” section—items of naming, follow verbal and written commands, writing a sentence and drawing a figure with intersections (9 points); Frontal Assessment Battery (FAB): This is a brief battery consisting of six subtests: similarities, lexical fluency, motor series, conflicting instructions, Go-No-Go test and prehension behavior). Each subtest can be scored from 0 to 3 points. The score is based on the sum of the six test scores.

To avoid a learning effect, some tests were applied in up to three different versions. The tests used in this study are among the most cited in INPH literature [17, 18].

### Matching procedure

Patients with IHPH and control subjects were matched using propensity scores (PS). Three variables were selected to estimate PS: age, sex and formal education. The propensity score was calculated using logistic regression and patients with IHPH were matched with controls using the nearest neighbor technique with a predefined caliper of 0.2 and considering the ratio 1:1. This matching procedure was performed using the MatchIt package for R.16.

### Statistics

A propensity score model was used to homogenize the INPH and the control group considering a logistic regression model and conditioning the variables of age, sex and formal education.

To analyze the effect of STT on cognitive test results of the INPH group in comparison with the control group, Z scores were calculated for each INPH patient regarding mean (M) and standard deviation (SD) of the control group. The Z scores of variables whose improvements were equivalent to smaller values, such as run time number of errors, variables dots, words and colors, and errors of the Stroop test, were multiplied by − 1 (inverted). Thus, in all variables, the highest values of the Z score corresponded to the participants’ best results.

Results of quantitative variables were described by means and standard deviations. Categorical variables were presented in frequencies and percentages. Comparison between the INPH and control groups, concerning categorical variables, was conducted using Fisher's Exact Test. Comparison of either two disease-defined groups or clinical factors, relative to quantitative variables, was conducted using either Student's t-test for independent samples or the non-parametric Mann–Whitney test. More than two groups were compared considering the nonparametric Kruskal–Wallis test. Normality condition was evaluated through the Kolmogorov–Smirnov test. Values of p < 0.05 indicate statistical significance. Data were analyzed using the IBM Corp. Released 2011. IBM SPSS Statistics for Windows, Version 20.0. Armonk, NY: IBM Corp.

After propensity score matching, from the 66 subjects with INPH and the 54 control subjects initially recruited, 61 INPH subjects and 42 control subjects remained for the statistical analysis. These groups were balanced regarding age (p = 0.056), sex (p = 0.231) and educational level (p = 0.549).

Statistical analysis was supervised by a professional statistician.

## Results

Regarding cognitive performance before the first LP, the INPH group scored lower than controls in every test except for the forward Digit Span Test. Values before and after the LP were also compared with control group scores (Table 3). The INPH group presented a similar score to the control group in the forward Digit Span Test in all steps of the serial drainage test. However, other measures of verbal memory, such as RAVLT—A1 and B1, improved after LP.

**Table 3:** INPH Pre-LP x post-LP1 and pre x post-LP2 cognitive scores, and control group cognitive scores

Variable	**n**	**BLP**			**ALP1**			**ALP2**		**CG**	
		**M (SD)**	p*	**n**	**M (SD)**	p**	**n**	**M (SD)**	p***	**n**	**M (SD)**
OR	61	9.6 (2.7)	** < 0.001**	59	9.9 (2.8)	** < 0.001**	55	9.9 (2.9)	0.001	42	11.7 (0.8)
MC	60	6.1 (2.3)	0.002	57	6.2 (2.2)	0.002	55	6.2 (2.2)	0.002	42	7.5 (1.1)
DS-For	59	5.4 (2.1)	0.707	55	5.7 (2.2)	0.885	54	5.7 (2.2)	0.606	42	5.6 (1.7)
DS-Back	59	3.3 (1.8)	0.003	55	3.4 (1.9)	0.026	54	3.4 (1.9)	0.015	39	4.2 (1.3)
FAR	58	20.7 (12.1)	** < 0.001**	54	22.4 (13.0)	**0.008**	52	23.9 (13.1)	0.051	41	29.6 (10.6)
Animals	58	9.3 (4.9)	** < 0.001**	53	9.2 (4.7)	** < 0.001**	53	9.6 (5.3)	** < 0.001**	41	13.9 (4.1)
A1	58	3.1 (1.4)	0.014	54	3.4 (1.7)	0.093	52	3.4 (1.6)	0.146	42	4.0 (1.8)
A5	57	6.0 (3.1)	** < 0.001**	54	6.6 (2.9)	** < 0.001**	52	6.0 (2.8)	** < 0.001**	42	9.6 (2.5)
Total	57	24.5 (10.4)	** < 0.001**	54	26.6 (10.5)	** < 0.001**	52	25.3 (10.6)	** < 0.001**	42	37.0 (10.3)
B1	56	2.7 (1.7)	0.010	53	2.7 (1.5)	0.007	51	3.3 (2.0)	0.293	42	3.7 (1.7)
A6	59	2.7 (1.7)	0.010	55	2.7 (1.5)	0.007	55	3.3 (2.0)	0.293	42	3.7 (1.7)
A7	58	3.2 (2.7)	** < 0.001**	56	3.0 (2.8)	** < 0.001**	55	2.5 (2.5)	** < 0.001**	42	6.7 (2.9)
S-Dots^a^	53	31.0 (24.8)	** < 0.001**	53	25.0 (19.3)	0.038	49	23.1 (16.8)	**0.014**	41	17.4 (5.3)
S-Word^b^	52	42.4 (31.6)	** < 0.001**	52	34.9 (25.8)	0.023	47	30.0 (19.0)	0.080	41	25.1 (9.1)
S-Colors^c^	52	58.6 (32.4)	0.003	49	52.8 (42.7)	0.255	47	46.1 (26.4)	0.479	39	42.8 (18.9)
S-Error^d^	46	8.1 (8.8)	** < 0.001**	46	6.2 (7.1)	0.002	39	4.7 (6.0)	0.092	41	2.3 (2.9)
CFT-cop	34	24.0 (9.4)	0.016	33	22.5 (9.5)	0.002	29	24.2 (9.4)	0.047	42	28.7 (7.1)
CFT- IR	34	7.5 (6.0)	**0.003**	31	10.0 (8.1)	0.159	27	11.5 (8.8)	0.389	42	12.2 (6.8)
CFT- DR	27	5.4 (4.5)	** < 0.001**	26	10.9 (11.8)	0.098	20	9.8 (9.0)	0.108	39	11.8 (6.0)

All values described in mean (M) and standard deviation (SD); n: number of individuals; BLP: step before lumbar punctures; ALP1: step after the first lumbar puncture; ALP2: step after the second lumbar puncture. Source tests: OR: orientation; MC: mental control; DS-FOR: forward digit span; DS-BACK: backwards digit span; FAR: verbal fluency test; RAVLT: Rey auditory verbal learning test; S-Dots^a^: Stroop Test—dots step; S-Word^b^: Stroop test – words step; S-Colors^c^: Stroop test- colors step; S-Error^d^: Stroop test – number of errors in the colors step; CFT-cop: Copy of Rey complex figure test; CFT-IR: Immediate reproduction of Rey complex figure test; CFT-DR: Delayed Reproduction of Rey complex figure test; *MMSE* Mini mental state examination, *FAB* frontal evaluation battery. P values: * non-parametric Mann–Whitney test, p < 0.05

The INPH group scored lower than controls in FAB and MMSE scales before LP and after the two LP remained lower than those of the control group (Mann–Whitney, p = 0.017 and p = 0.034, respectively).

Table 4 shows the comparisons among the three steps of the cognitive test for all tests applied during STT.

**Table 4:** Comparison of the three assessment moments of the INPH group

Original test	Variable	n	BLP	ALP1	ALP2	p*
			Mean ± SD		(Z Score)	
OR	Orientation	54	− 2.7 ± 3.5	− 2.4 ± 3.7	− 2.2 ± 3.8	0.018
MC	Mental control	53	− 1.3 ± 2.2	− 1.1 ± 2.1	− 1.2 ± 2.1	0.156
DS	Forward	51	− 0.1 ± 1.3	0.1 ± 1.3	0.1 ± 1.3	0.327
	Backward	51	− 0.7 ± 1.4	− 0.6 ± 1.4	− 0.6 ± 1.4	0.600
VF	FAR	28	− 1.2 ± 1.6	− 1 ± 1.8	− 0.9 ± 1.5	*0.053*
	Animals	49	− 1.1 ± 1.2	− 1.2 ± 1.1	− 1 ± 1.3	0.753
RAVLT	A1	50	− 0.5 ± 0.8	− 0.5 ± 0.8	− 0.4 ± 0.9	0.490
	A5	50	− 1.5 ± 1.2	− 1.2 ± 1.1	− 1.5 ± 1.1	0.306
	Total A1−A5	50	− 1.2 ± 1	− 1.1 ± 0.9	− 1.2 ± 1	0.113
	B1	49	− 0.6 ± 1	− 0.6 ± 0.8	− 0.3 ± 1.2	0.024
	A6	51	− 1.2 ± 0.9	− 1.3 ± 0.9	− 1.5 ± 0.8	0.081
	A7	51	− 1.3 ± 0.9	− 1.4 ± 0.9	− 1.5 ± 0.8	0.048
STROOP	S-Dots	45	− 2.2 ± 4.5	− 1.4 ± 3.3	− 1.2 ± 3.1	< 0.001
	S-Words	43	1.5 ± 3.3	1 ± 2.5	0.7 ± 2.1	0.031
	S-Colors	42	− 0.6 ± 1.6	− 0.5 ± 2	− 0.3 ± 1.4	0.541
	S-Errors	36	− 1.9 ± 3.1	− 1.4 ± 2.4	− 0.9 ± 2.2	0.010
CFT	CFT-Cop	26	− 0.6 ± 1.3	− 0.8 ± 1.4	− 0.8 ± 1.3	0.254
	CFT-IR	24	− 0.6 ± 0.9	− 0.4 ± 1.1	0 ± 1.3	0.006
	CFT-DR	15	− 1 ± 0.8	0 ± 2.3	− 0.4 ± 1.4	0.167
MMSE	MMSE	48	− 1.9 ± 2.6	− 1.6 ± 2.8	− 1.4 ± 3	0.021
FAB	FAB	28	− 1.2 ± 1.6	− 1 ± 1.8	− 0.9 ± 1.5	0.188

All values converted and described in Z scores. *SD* standard deviation, *n* number of individuals, *BLP* step before lumbar punctures, *ALP1* step after the first lumbar puncture,* post ALP2* step after the second lumbar puncture. Source tests: *OR* orientation, *MC* mental control, *DS* digit span, *VF* verbal fluency test, *FAR* lexical fluency; Animals: semantic fluency; RAVLT: Rey auditory verbal learning test; S-Dots: Stroop Test—dots step; S-Word: Stroop test – words step; S-Colors: Stroop test- colors step; S-Error: Stroop test – number of errors in the colors step; CFT-cop: Copy of Rey complex figure test; CFT-IR: Immediate reproduction of Rey complex figure test; CFT-DR: Delayed Reproduction of Rey complex figure test; *MMSE* mini mental state examination, *FAB* frontal evaluation battery. P values: * Non-parametric Friedman test, p < 0.05

Table 5 summarizes all variables with any significant improvement among the three testing steps, except for RAVLT-A7 (comparison between the moment before lumbar puncture and after the second lumbar puncture), which presented worsening.

**Table 5:** Analysis of the differences of three evaluation moments of the INPH group

Cognitive variable	Difference between steps			p
	A* scores	B* scores	C* scores	
	pre-LP x post-LP1	pre-LP x post-LP2	post-LP1 x post-LP2	
OR—orientation	0.121	0.004	0.177	0.018
MMSE	0.031	0.008	0.614	0.021
RAVLT—B1	0.711	0.028	0.011	0.024
RAVLT—A7*	0.153	0.014	0.290	0.048
ST—dots	p < 0.001	p < 0.001	0.855	p < 0.001
ST—words	0.069	0.010	0.422	0.031
ST—errors	0.241	0.002	0.053	0.010
CFT- IR	0.111	0.001	0.066	0.006

P values: * Non-parametric Friedman test, p < 0.05; Post-hoc analysis with comparisons of groups. LP: lumbar puncture. Variable with its respective test of origin: OR: orientation; MMSE: Mini Mental State Examination; RAVLT-B1: B list of the Rey verbal learning test; RAVLT-A7: late recall [20 min after the list A6] of the Rey verbal learning test; ST-dots: first step [dots] of the Stroop test in the University of Victoria version; ST-words: second step [words] of the Stroop test in the University of Victoria version; ST-errors: numbers of errors in the third step [colors] of the Stroop test in the University of Vitoria version; CFT-IR: Immediate reproduction [after 3 min] of the Rey complex figure test

An additional analysis was performed considering the previous history of associated clinical conditions of the INPH group, even if these conditions were not present for more than three years before the research period (Table 2). No differences were observed between subgroups with a history of previous depression, traumatic brain injury, or arterial hypertension. The stroke group presented better performance than the group without stroke in orientation (comparison between the moment before lumbar puncture and after first lumbar puncture) and the B1 memory RAVLT item (comparison between moment after first lumbar puncture and after second lumbar puncture; p = 0.034 and p = 0.047, respectively). All stroke individuals presented INPH symptoms for less than a year and did not report any cognitive permanent sequelae after the stroke event. Participants with diabetes presented greater mental slowness when compared with nondiabetic individuals (p = 0.029), even after the second LP.

## Discussion

INPH is one of the many diseases that can affect both motor and non-motor circuitry of the basal ganglia, and cause motor, autonomic, cognitive and behavioral symptoms [19]. Our study aimed to focus only on cognitive-behavioral manifestations of INPH. The STT protocol was carried out at a hospital ward, avoiding the need for commuting, an aspect that contributed to its acceptance by patients and family. Hospitalization allowed all stages of the procedure to be controlled regarding external interferences on test results.

Serial drainage testing resulted in lower morbidity than continuous lumbar drainage [8, 20]. Moreover, the second LP may increase the sensitivity of STT once detects more changes in cognitive variables, thus improving the method.

INPH subjects had lower performance than controls before LP in all tests regarding selective attention measures (words and colors in the Stroop test), distraction resistance (errors in the Stroop test), immediate and late visual memory (CFT). Similar results were described by Katzen et al*.* [21]. The exceptions to this finding in our study were the Digit Span Test. The fact that the Digit Span Scores were calculated forward and backward separately, probably triggered the differences regarding other studies [19, 22, 23], perhaps due to a ceiling effect in control individuals. However, other measures of supraspan, such as A1 and B1 of RAVLT, showed improvement.

The scores of the INPH group before LP were lower than the control group scores in both screening scales (MMSE and FAB) as previously observed by Katzen et al*.* [21] and Saito et al*.* [23]. Both scores remained lower after LPs, which reinforces the finding of severely compromised cognitive function in this population.

Eight cognitive items improved along with the STT, but the most paradoxical result was the decline of the RAVLT-A7 item, contrasting with the improvement seen in other tests. Serial Tap Test is an extensive protocol composed of several tasks applied to an elderly population. RAVLT-A7 item is the last phase carried out on the second day of examination and, for this reason, probably fatigue may have contributed to this result.

Significant improvement in A* scores (Table 5) probably reflect an enhancement in alertness [24]. The complex relationships between alertness and attention have been adequately discussed in the literature [25].

It seems likely that both the enhancement in alertness and the decompression of frontostriatal circuits promote a gradual improvement in many cognitive aspects, especially in the dots and words variables of the Stroop test, in which subjects reacted faster after LP. Participants also showed improvement in their ability to inhibit impulsive responses and to resist distractions (errors in the Stroop Test) [26]. Perhaps enhanced spatiotemporal perception revealed by the orientation test is also secondary to a better state of alertness. Isik et al. [27] performed serial punctures in the INPH patients group (mean duration interval of 7.4 ± 5.7 months between the first and second LP and a mean duration of 8.5 ± 3.8 months between second and third punctures). Each time, they tested these patients before LP and 24 h after. They found not a significant difference in the Stroop Test comparisons, at the first and second puncture moments. This result contrasts with what we found out. The first explanation is that our time interval between LP and the neuropsychological assessment was shorter (mean 4 h). Although INPH physiopathology is not so far well explained, immediate and delayed mechanisms may be involved. A decompression effect may be immediately releasing attentional circuits, promoting a cognitive enhancement in the alertness status. This aspect, however, does not appear to be the only mechanism involved in INPH cognitive dysfunction.

Several other hypotheses were formulated to explain the complex pathophysiology of this disease, some of which involve cerebral parenchyma and CNS blood vessels, accumulation of toxic metabolites in CSF, and transependymal CSF permeation with cell and axonal damage [15, 19, 28–32].

A similar effect on step A1 and B1 of RAVLT could be expected due to this gain in alertness since the structure of the two lists of words is similar. Despite the improvement seen in B1 (p = 0.024), the same was not found in A1 (p = 0.490). The structure of RAVLT may explain this difference since B1 is presented as a distraction element between the learning curve (A1-A5) and the immediate recall (A6) [33], whereas A1 is the first list presented in the test.

According to the theory of interference [12–14], forgetfulness can be understood as the interference of one memory over another. In RAVLT, a proactive interference (PI) is when prior learning affects later learning, but also a retroactive interference (RI) can occur, in which further learning affects the recovery of previously learned information. Thus, the improvement seen in B1 reflects a decrease in PI, but, in contrast, there was no change in RI (A6 – p = 0.081).

Time spent on the STT and the differences in the A*, B*, and C* scores indicated cognitive evolution over the two days of examination. Time reported being required for cognitive improvement after LP varies among authors from 30–60 min to a week [8, 34–36]. However, all cognitive studies regarding TT screened for changes after a single puncture [4, 6, 8, 9, 36–39]. The present study is so far the first to systematically use serial lumbar punctures and systematically retest cognition over steps and time. Significant improvement in several cognitive domains in such a short time interval, compared with those reported in previous literature, suggests that the changes detected were a result of repeated punctures rather than merely the passage of time. Therefore, according to our results, mental speed, the earliest improved function after LP, continues improving after the second LP. It is likely that other skills may improve because of mental speed increasing, e.g., phonetic, and lexical verbal fluency tasks. These kinds of tests depend on language and executive functions. NPHI is not presumed to affect directly cortical functions as language fluency, but to mechanically compress the periventricular frontostriatal circuits and to cause transependymal CSF leakage and parenchymal edema, affecting language pathways speed. Thereafter, the progressive release of this circuitry may ameliorate its functioning.

Some limitations of this study should be considered. No retest in the control group was performed. Comparisons between the INPH group after LP and the control group after mental function re-testing would clearly define whether the changes observed in INPH subjects were not a learning effect (LE). LE may depend on individuals’ characteristics as age and performance baseline [40]. A recent review [41] identified a lower LE in individuals with dementia as compared to normal aging individuals [42–45]. Our series of INPH individuals scored significantly below the normal aging group in all tests (except for the “digit span backward” subtest), a finding congruent with a cognitive decline. A single study about LE in INPH was published by Solana et al. [40] reported no learning effect in the INPH population when reapplying the same cognitive tests over four consecutive days. Our testing protocol started before Solana et al*.* [40] publication and has slight differences from their protocol, but the tests we have used are among the most used in INPH literature and there is a vast literature about patients' performance on them [17, 19, 21, 22, 35].

LE may depend on test characteristics as well, for example, a short-time-interval can result in a strong LE [40], and we sought for alternative testing to reduce the influence of this variable.

Lack of data on depression was also a gap in the study. However, self-evaluative scales have a limited effect on this population due to difficulties in differentiating depressive symptoms from frontal dysfunction. Kito et al*.* [46] observed that apathy is the most common neuropsychiatric disorder in this population and that this symptom has a high correlation with cognitive symptoms of the triad. Apathy is considered a symptom of somnolence-sopor-coma disorder (SSCD) [24, 47], as well as the emotional asthenic syndrome or apathetic-abulic syndrome [41]. The correlation between apathy and executive dysfunction has been attributed to the association of this symptom with INPH dysfunctional brain areas, such as the anterior cingulate cortex (ACC) and thalamus [48].

Another drawback is the lack of follow-up data after CSF shunting, and there is no doubt that this information is desired. To fill this gap, longitudinal information is being gathered to confirm the long-term improvement of the aforementioned cognitive aspects and their consistency over time.

## Data Availability

Not applicable.
